# Promoting psychosocial well-being following stroke: study protocol for a randomized, controlled trial

**DOI:** 10.1186/s40359-018-0223-6

**Published:** 2018-04-03

**Authors:** Marit Kirkevold, Line Kildal Bragstad, Berit A. Bronken, Kari Kvigne, Randi Martinsen, Ellen Gabrielsen Hjelle, Gabriele Kitzmüller, Margrete Mangset, Sanne Angel, Lena Aadal, Siren Eriksen, Torgeir B. Wyller, Unni Sveen

**Affiliations:** 1 Institute of Health and Society and Research Center for habilitation and rehabilitation services and models (CHARM), University of Oslo, P.O.Box 1130, Blindern, 0318 Oslo, Norway; 2 Inland Norway University of Applied Sciences, P.O.Box 400, 2418 Elverum, Norway; 3 Faculty of Health UIT, The Arctic University of Norway, Campus, Narvik, Norway; 4 Department of Geriatric Medicine, Oslo University Hospital, P.O box 4956, Nydalen, 0424 Oslo, Norway; 5 Institute of Public Health, Aarhus University, Hoegh-Guldbergs Gade 6 A, 8000 Aarhus, Denmark; 6 Hammel Neurorehabilitation and Research Centre, Voldbyvej 15 8450, Hammel, Denmark; 7 Norwegian National Advisory Unit on Ageing and Health (Ageing and Health), P. O. Box 2136, 3103 Tønsberg, Norway; 8 Institute of Clinical Medicine, University of Oslo, and Department. of Geriatric Medicine, Oslo University Hospital, P.O box 4956 Nydalen, 0424 Oslo, Norway; 9 Dept. of Geriatric Medicine, and Dept. of Physical Medicine and Rehabilitation, Oslo University Hospital, P.O box 4956 Nydalen, 0424 Oslo, Norway

**Keywords:** Psychosocial rehabilitation, Stroke, Dialogue-based, Supportive care, Randomized controlled trial, Process evaluation, Implementation adherence, Intervention fidelity, Aphasia

## Abstract

**Background:**

Stroke is a major public health threat globally. Psychosocial well-being may be affected following stroke. Depressive symptoms, anxiety, general psychological distress and social isolation are prevalent. Approximately one third report depressive symptoms and 20% report anxiety during the first months or years after the stroke. Psychosocial difficulties may impact significantly on long-term functioning and quality of life, reduce the effects of rehabilitation services and lead to higher mortality rates. The aim of the study is to evaluate the effect of a previously developed and feasibility tested dialogue-based psychosocial intervention aimed at promoting psychosocial well-being and coping following stroke among stroke survivors with and without aphasia.

**Methods:**

The study will be conducted as a multicenter, randomized, single blind controlled trial with one intervention and one control arm. It will include a total of 330 stroke survivors randomly allocated into either an intervention group (dialogue-based intervention to promote psychosocial well-being) or a control group (usual care). Participants in the intervention group will receive eight individual sessions of supported dialogues in their homes during the first six months following an acute stroke. The primary outcome measure will be psychosocial well-being measured by the General Health Questionnaire (GHQ). Secondary outcome measures will be quality of life (SAQoL), sense of coherence (SOC), and depression (Yale). Process evaluation will be conducted in a longitudinal mixed methods study by individual qualitative interviews with 15–20 participants in the intervention and control groups, focus group interviews with the intervention personnel and data collectors, and a comprehensive analysis of implementation fidelity.

**Discussion:**

The intervention described in this study protocol is based on thorough development and feasibility work, guided by the UK medical research council framework for developing and testing complex interventions. It combines classical effectiveness evaluation with a thorough process evaluation. The results from this study may inform the development of further trials aimed at promoting psychosocial well-being following stroke as well as inform the psychosocial follow up of stroke patients living at home.

**Trial registration:**

NCT02338869; registered 10/04/2014 (On-going trial).

## Background

Stroke is a major global health problem [1]. Psychosocial well-being is frequently threatened following stroke. Depressive symptoms, anxiety, general psychological distress and social isolation are prevalent [2]. Approximately one third report depressive symptoms and 20% report anxiety during the first months and depression may be present years after the stroke [3–5]. Psychosocial difficulties may impact significantly on long-term functioning and quality of life [4, 6], reduce the effects of rehabilitation services and lead to higher mortality rates [7].

A large number of studies have explored possible interventions to prevent and/or treat psychosocial problems [7–10], but results have generally been disappointing. Pharmacological treatment may be effective in treating post-stroke depression, but not in preventing it. Furthermore, antidepressants may have adverse effects in persons with stroke and should be used with care [7, 11]. Consequently, there is a need for developing alternative interventions. So far, psychosocial interventions have had modest effects; however, the findings conclude that information, emotional support, practical advice and motivational support are important [8, 10, 12, 13]. It remains unclear how the different elements of the interventions contribute to positive outcomes and which elements work best at different stages and among different subgroups [8, 10, 12]. Few studies have provided adequate theoretical accounts of the mechanisms assumed to contribute to positive outcomes [8, 10, 12].

Aphasia affects about one third of the stroke population [14] and 40% continues to have significant language impairment at 18 months post-stroke [15]. Language is the most important tool for human interplay, social participation and community. Aphasia is associated with major disruptions of everyday life and affects all dimensions of quality of life [16, 17]. Persons with aphasia (PWA) are especially prone to psychosocial problems, such as anxiety and depression, threatened identity, changes in interpersonal relationships, reduced social networks, social isolation, unemployment and abandonment of leisure activities [18–23]. The emotional and psychosocial factors have a marked impact on recovery, the psychosocial adjustment process, and the response to rehabilitation [24, 25]. Nevertheless, psychosocial interventions targeting this group are sparse and access to such services very limited.

The incidence of stroke increases dramatically with increasing age [1]. However, stroke may appear at any age. Among younger work-aged stroke survivors (aged 18–67 years), the psychosocial factors appear to have at least as great an impact on life after stroke as the physiological consequences [26–28]. Despite the potential serious consequences among younger persons, few intervention studies have sought to develop psychosocial interventions tailored to their specific needs.

Registered nurses (RNs) are among the members in the rehabilitation team who are expected to address the psychosocial needs of patients through providing support and guidance to improve coping [29, 30]. Nevertheless, only a few nursing interventions have been developed, specifically addressing the psychosocial well-being of stroke survivors [31–34]. These are promising, but have mostly been conducted by hospital-based staff. However, a major thrust of the rehabilitation occurs in the community. Consequently, effective interventions for primary health care are needed. Nurses are the most numerous professionals within this sector and are frequently the front line workers providing care to stroke survivors. Other health care professionals as well, including occupational therapists (OTs), are responsible for promoting coping and adjustment to the consequences of stroke [35, 36]. However, few interventions delivered by primary health care professionals have been developed and tested.

To address this void, we have developed and initially tested a dialogue-based psychosocial intervention primarily carried out in primary care, aimed at supporting the coping and life skills of stroke survivors [37]. In this work, we applied a development and testing approach consistent with the recommended framework for developing and evaluating complex interventions proposed by the UK Medical Research Council [38, 39]. This framework describes the development and testing of complex health interventions in four interacting phases, from the initial development phase, through the ‘modeling’ and ‘exploratory trial’ phases into the ‘RCT’ phase and finally the ‘long-term implementation’ phase. In our previous studies, we have completed the first three phases. In the first two phases, we reviewed relevant research and theory to develop an empirical- and theory-based intervention [37]. In the exploratory trial phase, we used a multiple case study approach, drawing on different data sources to explore if the intervention was found useful by twenty five stroke patients [40].

The participants in the exploratory trial/feasibility study found the content and process of the intervention relevant. The participants underscored the benefits of being supported through a difficult time, provided a chance to tell and (re)create their story and being supported in their attempts to cope with the situation. Receiving psychological support and motivation to move on during the difficult adjustment process, and exchange of knowledge and information were also experienced as beneficial and important by the participants [41–44]. The aphasia group emphasized the importance and experienced benefit of receiving language support through the opportunities to speak and being supported in communicating about their experiences by a knowledgeable dialogue partner [41, 42]. The study provided initial support for the usefulness of the psychosocial intervention and suggested aspects that needed further consideration and development. In particular, the intervention should be further developed for persons with aphasia. In addition, the younger participants (i.e. people in working-age) emphasized the need for a psychosocial program that specifically addressed their particular challenges and needs as carers for children, breadwinners in their families and as employees struggling to return to work [43]. Based on these findings, testing in a larger, controlled trial is warranted.

In the current study, we use the same content, structure and process that were tested in the initial exploratory trial/feasibility study. However, based on the findings from the preliminary work, we have adjusted the intervention to accommodate the weaknesses and challenges uncovered in the exploratory trial/feasibility study.

### Aims and hypotheses

The primary aim of the study is to determine whether a previously developed and feasibility-tested dialogue-based psychosocial intervention promotes psychosocial well-being and coping following stroke among stroke survivors with and without aphasia, compared to usual care. We will test the following hypotheses:

1. *Primary outcome:* Stroke survivors with and without aphasia in the intervention group will experience significantly higher levels of psychosocial well-being and lower levels of depressive symptoms and anxiety (measured by GHQ-28) than stroke survivors in the control group at 6 and 12 months post stroke
2. *Secondary outcomes:* Stroke survivors with and without aphasia in the intervention group will experience significantly higher levels of sense of coherence (measured by SOC-13) and health-related quality of life (measured by SAQOL-39) than stroke survivors in the control group at 6 and 12 months post stroke

The secondary aim of this study is to conduct a process evaluation in order to understand the change mechanisms of the intervention and how these impact on the participants’ study outcomes. To improve our ability to interpret the study outcomes, the process evaluation will assess:

1. How participants in the intervention and control group experience their adjustment process following the stroke and their participation in the study
2. How the personnel delivering the intervention experiences their role in the intervention delivery and the participants’ responsiveness to the intervention
3. How the data collectors experienced the data collection interviews with the participants, including the acceptability and/or difficulties in applying the instruments in this population
4. Implementation fidelity and intervention adherence throughout the study

## Methods

### Design

The study is a prospective multicenter randomized controlled trial to evaluate the effectiveness of a dialogue-based longitudinal intervention study the first six months following a cerebrovascular stroke for persons with and without aphasia. The trial has one intervention and one control arm. The process evaluation is a longitudinal mixed-methods study. The protocol is prepared according to the Standard Protocol Items: Recommendations for Interventional Trials (SPIRIT).

### Setting

The study is primarily conducted in community care settings in Norway. The intervention is mainly delivered in the homes of the participants. However, in the initial phases, the dialogue-based sessions may be conducted in other settings if necessary, such as in a hospital, at a rehabilitation unit or another place where the participants find themselves. The intervention is delivered by specially trained nurses and occupational therapists.

### Participants

#### Inclusion and exclusion criteria

This RCT study includes stroke survivors meeting the following inclusion criteria: Being adults over 18 years of age, suffered an acute stroke within the last month prior to inclusion, medically stable, sufficient cognitive functioning to participate (assessed by their physician/stroke team), interested in participating, able to understand and speak Norwegian, and able to give informed consent.

Exclusion criteria include moderate to severe dementia, serious somatic or psychiatric disease as these are assumed to impact on the ability to participate in the intervention. All participants are screened for aphasia using the Ullevaal Aphasia Screening Test (UAS) [41], and a speech therapist will be consulted if needed. Persons will be excluded if they have significant impressive aphasia or severe expressive aphasia, but will otherwise be included.

### Sample size calculations

The study’s sample size was calculated based on the primary outcome measure GHQ-28, which has been used in a comparable trial [31, 32]. Following Watkins et al.’s results, we deemed an odds ratio of 1.6 or higher between-groups (intervention/control) with normal mood after 6 and 12 months to be clinically relevant. The calculations are based on a repeated measures logistic regression model for the output variable “normal mood” (GHQ-28 < 5) with two measurements for each patient (i.e. one at 6 months and one at 12 months), with one binary input variable specifying group allocation. Based on 80% power across both time points, the calculated sample size of this study is 300 participants, 150 in each arm of the study. To allow for a 10% dropout rate, we have inflated the number to 330 participants in total.

### Study procedures

#### Recruitment and consent

Participants are recruited from 11 acute stroke units or rehabilitation units in university hospitals and other local hospitals providing acute care in Norway. Participants are identified by specifically trained clinical staff in the participating units, based on the stated inclusion and exclusion criteria following the recruitment protocol of the study. Recruitment occurs when the patient is medically stable and considered ready for receiving information about the study. Eligible patients, who have suffered an acute stroke within the last four weeks, will receive oral and written information and be invited to enter the study. Potential participants who are not ready to decide whether they will participate or not at this early stage, will be given information and asked if they can be contacted at a later stage. The written information sheet and consent form have been developed to accommodate aphasia and have been approved by the Regional Medical Ethics committee and the Data Protection Officer at the participating hospitals. Recruitment will occur over a three-year period.

#### Randomization and blinding

A computer-generated block randomization procedure created by a statistician independent of the research group is applied. The participants are randomized in blocks of 10 to minimize allocation bias, and to ensure an equal group size in intervention and control groups. The randomization is stratified by study center. Opaque randomization envelopes with a five-digit patient identification number printed outside and a note specifying intervention or control inside, are prepared according to the computer-generated randomization lists by an assistant independent of the research group. Two regional study coordinators carry out the randomization process following a completed baseline assessment. To ensure blinding of the assessors, group allocation is communicated solely to the patient him or herself and the health care professional delivering the intervention when the patient is randomized to the intervention group. To maintain blinding at follow up assessments, a text message is sent from the study coordinator to each participant prior to each assessment reminding them not to reveal their group allocation to the assessor.

### The intervention: Promoting psychosocial well-being following stroke

#### Theoretical perspectives underpinning the intervention

The overall goal of the intervention is to promote psychosocical well-being, defined as: (a) a basic mood of contentment, pleasure and well-being and the absence of sadness or a feeling of emptiness, (b) participation and engagement in meaningful activities beyond oneself, (c) good social relations and a feeling of loving and being loved in mutual relation(s), and (d) a self-concept characterized by self-esteem, self-acceptance, usefulness and belief in one’s own abilities [42]. Each of these dimensions will be addressed in the dialogues.

Experiences of chaos and a lack of control are major threats to well-being following stroke. Antonovsky’s theory connects health and well-being to the experience of a sense of coherence in life (SOC). SOC is promoted by experiencing life events as comprehensible (cognitive), manageable (instrumental/behavioural) and meaningful (motivational) [43, 45, 46]. SOC is seen as an essential intermediate goal for promoting psychosocial well-being [37].

To promote SOC, we draw on narrative theory [47, 48], which emphasises that human beings create meaning in their lives through the stories they tell. Through stories, people seek to negotiate a position within a given social context that gives meaning, direction, identity and value to their lives [49, 50]. Research suggests that telling one’s story is a fundamental need following a traumatic event and that this may promote well-being in and of itself [51, 52]. We assume that being encouraged and supported to tell one’s story, receiving response from others and experiencing that stories are shared would stimulate reflection and adjustment and strengthen identity, self-understanding and self-esteem.

People suffering from aphasia are restricted in their natural abilities to tell their stories [53, 54]. The method “Supported Conversation for Adults with Aphasia” assigns a particular responsibility for facilitating social interactions to the person without communication difficulties and provides a number of different techniques that may enhance communication and understanding in dialogues with PWA [55].

To promote coping and development of new life skills, we apply ideas from guided self-determination [56], an approach inspired by empowerment philosophy. It highlights the importance of being in control of one’s own recovery- and adjustment process. In this approach, the role of the health care professional is conceptualized as being a “supporter” or “coach” rather than a “carer” or “therapist”. The participants are in charge of the dialogues in the sense that they decide what to focus on in each encounter, whereas the health care professional follows the participants’ lead. At the same time, the structure of the intervention, with the guiding topical outline of each encounter and supporting work sheets, provide a framework for the dialogues that the participant and health care professional may follow or deviate from [37].

### The intervention

**Fig. 1:**
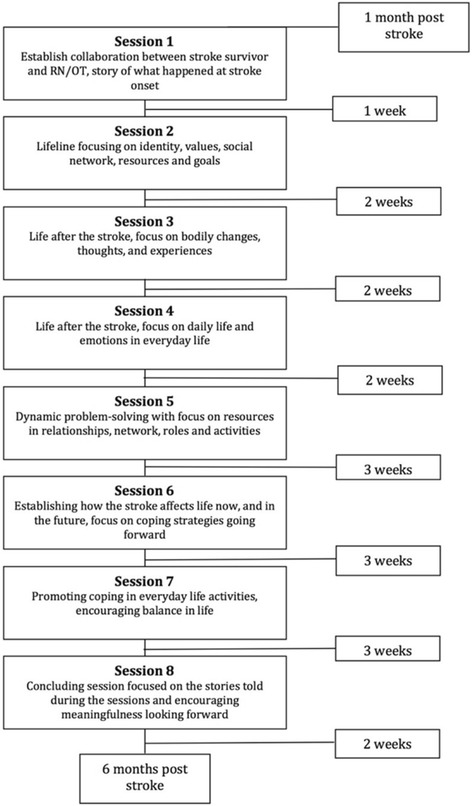
Flowchart of intervention with main content in each session

The intervention consists of 8 one to one and a half hour dialogue-based sessions between the stroke survivor and a specially trained health professional (RN or OT). Each meeting has a guiding topical outline, which addresses significant issues described in the research literature (e.g. bodily changes, emotional challenges, personal relations, daily life issues, meaningful activities, existential issues, important values etc.) (Fig. 1).

In the sessions, work sheets developed to support the dialogues are used. The work sheets consist of drawings, figures, unfinished sentences, and key words that point to the topic that the participants are invited to address. The work sheets are adapted to persons with aphasia. The sessions are in part carried out as open dialogues, where the participants are invited to tell about issues that are important to them at the time, and in part structured by the work sheets developed for the particular meeting. If the participant initially introduces a topic that is different from the topic suggested for the particular session, the health care professional changes the planned order of topics, i.e. by using work sheets from other planned sessions. In this way, the intervention is made flexible to meet the individual participant’s needs. The first session occurs approximately between 4 and 8 weeks post stroke, the last is conducted before the 6 months post-stroke time point (Fig. 1). The intervention is offered to the participants during the period when the recovery- and adjustment process is assumed to be most challenging. The sessions take place at times of increased vulnerability due to known transition points (i.e. at discharge, when physical improvement slows down, when people tend to assume new challenging roles or activities etc.). The number of sessions (8) is chosen in an attempt to balance the ideal with the realistic (i.e. as few encounters as possible but enough to provide adequate support).

### Control group

The control group receives treatment as usual and no intervention beyond participation in the assessment interviews at 1, 6, and 12 months. As participants are recruited from a variety of settings (acute stroke care and rehabilitation units), we anticipate some variation in the usual care provided. Provision of rehabilitation services is the responsibility of the community health care services in Norway, and this adds to the anticipated variability in services. Systematic psychosocial rehabilitation and support is not implemented in community health care services in Norway. However, adoption of the National stroke treatment guidelines in the acute phase of stroke (based on international stroke treatment guidelines) is high in Norway. To control for the potential variability in “usual care”, we collect data on the types of rehabilitation services received in both arms of the study.

### Outcome measurements

The primary outcome is psychosocial well-being as measured by The General Health Questionnaire (GHQ-28). The GHQ was developed by Goldberg [57] and has been translated into Norwegian by Malt and colleagues [58]. The GHQ-28 has been used in other trials involving psychosocial stroke interventions [31, 32, 59] and was also used in Hilari et al.’s study of SAQOL-39 [44], which will facilitate comparison.

The Stroke Aphasia Quality of Life scale (SAQOL-39) addresses general dimensions of health-related quality of life (HRQOL). It is based on the Stroke-specific Quality of Life scale (SS-QOL) and was adjusted for persons with aphasia. It has been found to be valid and reliable in the general stroke population and to be sensitive to change [44, 60]. SAQOL 39 was tested in the exploratory trial for appropriateness and was found to function adequately.

Sense of Coherence (SOC-13), has been translated into Norwegian and previously been applied in studies focusing on psychosocial well-being [61–64].

NIHSS is a well-established assessment for measuring stroke severity used in numerous stroke studies [65].

Yale is a one item instrument measuring the presence or absence of depression as experienced by the person. It has been validated as a measurement for depression in stroke patients [66, 67].

Lee Fatigue Severity Scale (5 items) and Fatigue Questionnaire (2 items) have been chosen to measure fatigue in this study [68, 69].

The Ullevaal Aphasia Screening test (UAS) is a quick and simple aphasia screening which can be used by health professionals other than speech therapists to discriminate between aphasia and normal language. It has been shown to be reliable in several studies [41, 70]. The instruments and related constructs are summarized in Table 1.

**Table 1:** Overview of instruments and related constructs

Construct	Instrument	Domains
Psychosocial well-being	The General Health Questionnaire-28 (GHQ-28)	28 item general scale measuring emotional distress. Four subscales (somatic symptoms, anxiety/insomnia, social dysfunction and serious depression
Psychosocial well-being	The Stroke and Aphasia Quality of Life scale (SAQOL-39)	Disease-specific quality of life scale, measures patient’s perspective of stroke’s impact on ‘physical’, ‘psychosocial’ and ‘communication’ domains.
Sense of coherence	Sense of Coherence scale (SOC-13)	Self-report questionnaire, 13 components, measuring the main concepts in the SOC theory; coherence, meaningfulness and manageability. 13 items scored on a Likert scale, ranging from 1 to 7. Higher scores indicate a stronger SOC.
Depression and anxiety	The Yale Brown single item questionnaire (Yale)	One yes/ no question
Fatigue	Lee’s Fatigue scale-5 (Lee-5)	A 0–10 scale assessing symptoms of fatigue
Fatigue	Fatigue Questionnaire-2 (FQ-2)	One yes/ no question, If yes; length of symptoms.
Demographics		Age, gender, ethnic background, education, type of work/studies, marital status, living condition, family/network, place of living (urban/rural)
Medical information		Time of stroke, type / localization of stroke, type of medical treatment after stroke, medication, other chronic diseases, earlier depression / mental disorders, rehabilitation services provided, type and amount of health care/practice assistance provided in the community.
Stroke severity	National Institutes of Health Stroke Scale (NIHSS)	A questionnaire used by healthcare providers to objectively quantify the impairment caused by a stroke.
Aphasia	The Ullevaal Aphasia Screening Test (UAS)	Screening for aphasia.

All instruments will be administered at T1 (at 1 month post-stroke and before randomization and potential initiation of the intervention), at T2 (6 months after stroke, approximately two weeks after the end of the intervention), and at T3 (12 months after stroke / 6 months after the end of the intervention). The instruments will be administered by data collectors with a health care background (RN and OT) using a personal interview format. The assessor is blinded to group allocation at each data collection point. At T0 (at hospital / rehabilitation unit) medical information about the stroke and stroke severity (NIHSS-score) will be collected. Data and all appropriate documentation will be stored for a minimum of 5 years after the completion of the trial, including the follow-up period. The Standard Protocol Items: Recommendations for Interventional Trials (SPIRIT) Flow diagram showing the schedule for enrolment, interventions and assessments is provided in Table 2.

**Table 2:** Schedule of enrolment, intervention, and assessments (SPIRIT)

Time point	Enrolment -t1 4–8 weeks post-stroke	Data collection t1 at time of enrolment	Allocation Immediately following data collection t1	Intervention period 4 weeks-6 months post-stroke	Data collection t2 6 months post-stroke	Data collection t3 12 months post-stroke
Enrolment						
Eligibility screen	X					
Informed consent	X					
Intervention						
Randomization			X			
Intervention: Psychosocial dialogues				X		
Control: Usual care				X		
Assessments						
Primary outcome:						
GHQ 28		X			X	X
Secondary outcomes:						
SAQOL 39		X			X	X
SOC 13		X			X	X
Yale		X			X	X
Characteristics of sample:						
Demographics		X			X	X
Lee 5		X			X	X
FQ 2		X			X	X
Medical information	X	X			X	X
NIHSS	X					
UAS		X				

### Statistical analysis

Data analysis will be conducted as intention-to-treat. Missing data will be imputed using multiple imputation technique [71]. All analyses will be performed using statistical software such as IBM SPSS or R. For group comparisons of individual variables, categorical variables will be analyzed using chi-squared tests, and continuous variables will be analyzed using t-tests or Mann-Whitney U tests (for two-group comparisons) and F-tests or Kruskal-Wallis tests (for more than 2 groups). The primary statistical analysis will be a repeated measures logistic regression model for the dichotomized output variable “normal mood yes/no” (GHQ-28 < 5 versus GHQ-28 ≥ 5) with two measurements for each patient (i.e. one at 6 months and one at 12 months), with one binary input variable specifying group allocation. Independent variables will include demographic variables, stroke type and severity measurements, rehabilitation service use and relevant medical variables. Medical and demographic variables will be included in the data analysis model to control for group differences at baseline. Secondary analyses, e.g. sub-group analyses, will be conducted with primary, secondary and process outcomes. The psychometric properties of GHQ-28 will be examined to confirm stability of dimensions in a Norwegian general stroke population across time points. All statistical tests will be performed as two-sided tests with a significance level of 0.05.

### Program assessment and treatment fidelity/process evaluation

#### Training and supervision

To promote high fidelity in intervention delivery, a 3-day training course has been developed to ensure intervention delivery according to protocol. The 3-day course is designed as an interactive training consisting of a range of lectures combined with practical training exercises, group reflection and discussions, and individual reading assignments. The training will give an in-depth introduction in guided self-determination, supported conversations for adults with aphasia, and cover all the theoretical underpinnings of the intervention (Table 3). The participants are provided with relevant research articles, books, and book chapters that provide updated knowledge on psychosocial issues following stroke, outline the theoretical underpinnings of the intervention, and provide guidance in the approaches chosen for the intervention.

**Table 3:** Overview of components of the 3-day training course

Main component	Lecture topic	Type of training
Understanding stroke survivor’s every-day challenges	Living with stroke	Educational video, group discussion
Theoretical underpinnings of the intervention	Introduction to the intervention’s essential ideas and philosophy underpinning the intervention	Lecture
	Psychosocial issues following stroke	Lecture
	Understanding challenges and changes for work-aged stroke survivors	Lecture
	Bodily changes following stroke	Lecture
	Gendered perspectives on stroke	Lecture
	Aphasia and living life with language impediments	Lecture
	The aphasic storyteller	Lecture
	Supported conversations for adults with aphasia	Lecture
	When things become incomprehensible: Cognitive and other invisible changes following stroke	Lecture
	Ethical considerations in the role as guide	Lecture
	Guided self-determination and the role as guide using the intervention’s metaphor	Lecture
Guided self-determination	Presentation of the intervention’s work sheets	Lecture
	Presentation of the intervention’s work sheets	Practical exercise, roleplay, group discussion
Supported conversation for adults with aphasia	Supported conversations for adults with aphasia	Practical exercise
	Using the work sheets and different approaches to communication	Lecture, practical exercise
Documentation	Using log notes	Lecture, group discussion
General discussion and reflection	Throughout the program	Group discussion

Health care professionals with knowledge and experience in working with stroke patients,

primarily nurses and occupational therapists, complete the 3-day training course to be certified to deliver the intervention. Intervention personnel writes a log, using a standardized format, to document how each session in the intervention is conducted, any problems encountered and how they deal with these. In addition, group supervising seminars for the intervention personnel are arranged throughout the study period. The seminars are led by members of the research team. These seminars provide an arena for exchange of experiences between intervention personnel and between intervention personnel and the research team. The purpose is to discuss any difficulties in the intervention encounters and support the intervention staff in implementing the intervention according to protocol. Sharing of experiences through storytelling and group reflection and discussions are the main mode of communication in the seminars. The seminars are an arena for guidance and supervision for the intervention personnel, and it allows the research team to uncover potential needs for reinforcement of training to promote intervention fidelity. Individual supervision is provided as needed between group supervision seminars.

Data collectors with a health care background (RN or OT) collect data for the study. They receive a written data collection procedure training protocol and individual training in administering each of the outcome measures. The data collectors are supervised to ensure that data is collected in a consistent manner throughout the study.

#### Process evaluation

The process evaluation of this study is guided by the process evaluation framework outlined by the MRC Population Health Sciences Research Network (PHSRN) [72]. As part of this evaluation, 15–20 participants from the intervention and control groups are invited to participate in individual qualitative interviews after study completion (12 months post stroke). The purpose of these interviews is to gain an in-depth understanding of the participants’ experiences with the adjustment process following the stroke, and their experiences with participating in the intervention or control group and assessment interviews. The interview approach is based on Ricoeur’s phenomenological hermeneutics [73] and the data will be analyzed according to Ricoeur’s interpretation theory [74].

Post-intervention focus groups will be conducted with intervention personnel following the completion of all intervention programs. The purpose of the focus groups is to ascertain an in-depth understanding of the intervention personnel’s experiences with delivering the intervention, their impression of participant responsiveness to the intervention, and to uncover potential threats and facilitators to implementation adherence fidelity.

Focus group interviews with data collectors conducting the data collection interviews will be conducted following completion of all data collection. The purpose of the focus group interviews will be to gain an in-depth understanding of the data collection interviews from the data collectors’ perspective and to assess the suitability of the chosen outcome measures. Thematic content analysis will be applied to the focus group interview data [75].

Detailed enrolment records, attrition protocol, and intervention protocols are logged to facilitate quantitative analysis of implementation fidelity and intervention adherence [76, 77]. Descriptive statistical analyses will be applied to the quantitative data with the purpose of describing the study’s implementation adherence. Furthermore, quantitative data from these protocols will be used to construct an independent variable of intervention adherence (fidelity) to be included in the outcome analyses of the study.

The attrition rate is expected to vary between the intervention and control groups due to the relative extensiveness of the intervention. We will attempt to identify key predictors of attrition status (i. e baseline stroke severity and demographic characteristics). The attrition protocol will be analyzed to inform the process evaluation with regards to possible adjustments in future studies.

## Discussion

A stroke may create a number of burdensome psychosocial difficulties and many stroke survivors report unmet support needs, particularly upon discharge from the specialized stroke care in the hospital [1–5]. Nevertheless, few effective interventions aimed at promoting coping and psychosocial well-being exist to support the stroke survivor, and their family, once they return to the community. Therefore, conducting well designed intervention studies are needed In this study, we test the effectiveness of a theoretically and empirically informed intervention, developed within the framework of complex interventions proposed by the UK medical research council [39]. Thorough feasibility testing prior to the trial, have helped inform the current trial [41–43] and improved the methodology as well as the intervention itself.

A major strength in this study is the combination of classical RCT methodology with a thorough process evaluation to enable us to evaluate the implementation fidelity and intervention adherence and be better equipped to interpret our findings. Furthermore, by recruiting participants from 11 hospitals and include close to thirty trained intervention staff to deliver the intervention, our trial will mirror the “real world” of stroke follow up care with its inherent variability in terms of both acute and rehabilitation services provided. This will strengthen the trials’ external validity. Because the intervention may be delivered by community-based nurses, and other health care professionals with limited additional training, the intervention will be easier to implement in community care, should it prove effective.

Despite the careful planning and feasibility testing before commencing the trial, there are a number of risks as well. These include the dependency of a large number of clinical staff and hospitals managers at a number of hospitals to ensure consistent recruitment and a great number of intervention staff, which may introduce variability in the delivery of the intervention. Furthermore, many researchers and research assistants are involved in data collection, which again may introduce inconsistency in data collection. To address these risks, we have developed a strong team of researchers with regular meetings, stringent protocols to ensure consistency, through training and supervision of all involved and two designated study coordinators responsible for one geographical area each. One designated project leader and a multidisciplinary team of senior researchers experienced in conducting trials will also monitor the trial to ensure that the study protocol is followed and/or address any problems that we might encounter.

### Trial status

Patient recruitment to the trial has ended, but recruitment to the process evaluation is ongoing at the time of manuscript submission. Data collection will continue to the end of June 2018.
